# Correlation between the Uptake of ^18^F-Fluorodeoxyglucose (^18^F-FDG) and the Expression of Proliferation-Associated Antigen Ki-67 in Cancer Patients: A Meta-Analysis

**DOI:** 10.1371/journal.pone.0129028

**Published:** 2015-06-03

**Authors:** Sheng-ming Deng, Wei Zhang, Bin Zhang, Yin-yin Chen, Ji-hui Li, Yi-wei Wu

**Affiliations:** 1 Department of Nuclear Medicine, The First Affiliated Hospital of Soochow University, Suzhou, China; 2 School of Radiation Medicine and Protection, Medical College of Soochow University, Suzhou, China; 3 Key Laboratory of Nuclear Medicine, Ministry of Health, Jiangsu Key Laboratory of Molecular Nuclear Medicine, Jiangsu Institute of Nuclear Medicine, Wuxi, China; 4 Department of Radiology, The First Affiliated Hospital of Soochow University, Suzhou, China; Fondazione IRCCS Istituto Nazionale dei Tumori, ITALY

## Abstract

**Objective:**

To study the correlation between ^18^F-FDG uptake and cell proliferation in cancer patients by meta-analysis of published articles.

**Methods:**

We searched PubMed (MEDLINE included), EMBASE, and Cochrane Database of Systematic Review, and selected research articles on the relationship between ^18^F-FDG uptake and Ki-67 expression (published between August 1, 1994-August 1, 2014), according to the literature inclusion and exclusion criteria. The publishing language was limited to English. The quality of included articles was evaluated according to the Quality Assessment of Diagnosis Accuracy Studies-2 (QUADAS-2). The correlation coefficient (r) was extracted from the included articles and processed by Fisher's r-to-z transformation. The combined correlation coefficient (r) and the 95% confidence interval (CI) were calculated with STATA 11.0 software under a random-effects model. Begg's test was used to analyze the existence of publication bias and draw funnel plot, and the sources of heterogeneity were explored by sensitivity and subgroup analyses.

**Results:**

According to the inclusion and exclusion criteria, 79 articles were finally included, including 81 studies involving a total of 3242 patients. All the studies had a combined r of 0.44 (95% CI, 0.41-0.46), but with a significant heterogeneity (I^2^ = 80.9%, *P*<0.01). Subgroup analysis for different tumor types indicated that most subgroups showed a reduced heterogeneity. Malignant melanoma (n = 1) had the minimum correlation coefficient (-0.22) between ^18^F-FDG uptake and Ki-67 expression, while the thymic epithelial tumors (TETs; n = 2) showed the maximum correlation coefficient of 0.81. The analytical results confirmed that correlation between ^18^F-FDG uptake and Ki-67 expression was extremely significant in TETs, significant in gastrointestinal stromal tumors (GISTs), moderate in patients with lung, breast, bone and soft tissue, pancreatic, oral, thoracic, and uterine and ovarian cancers, average in brain, esophageal and colorectal cancers, and poor in head and neck, thyroid, gastric and malignant melanoma tumors. Subgroup analysis indicated that positron emission tomography (PET) or PET/CT imaging technology or Ki-67 and standardized uptake value (SUV) measurement technology did not significantly affect the results of r values, and Begg's test showed no significant publication bias.

**Conclusion:**

In cancer patients, ^18^F-FDG uptake showed a moderate positive correlation with tumor cell proliferation. Different tumor types exhibited varied degree of correlation, and the correlation was significant in TETs and GSTs. However, our results need further validation by clinical trials with a large sample of different tumor types.

## Introduction

Higher cell proliferation rate is one of the poor prognostic factors of cancer patients. Currently, various methods can be used to evaluate tumor cell proliferation, and immunohistochemical determination of the expression of the nuclear marker of proliferating cells, Ki-67, is considered to be a reliable means to monitor cell proliferation. Ki-67 is a cell cycle-related protein, expressed in each but the G0 phase of cell cycle [1]. The expression of Ki-67 in poorly differentiated carcinomas is significantly higher than that in well-differentiated ones, and tumors with higher Ki-67 expression display increased invasiveness. However, the expression of Ki-67 in different tumor regions is not completely uniform. Therefore, use of a small tumor block to detect the expression of Ki-67 may not be accurate and thus have certain application limitations [2].

PET is a molecular imaging technique that can noninvasively evaluate a variety of human physiological processes, including cell metabolism, angiogenesis, receptor expression, drug uptake, cell proliferation, etc. ^18^F-FDG is one of the most commonly used PET radionuclide imaging agents, widely used in cancer diagnosis, disease staging, biopsy target delineation, efficacy assessment, etc. However, its application has some limitations. For example, ^18^F-FDG is not a tumor-specific imaging agent, and it can also be absorbed by certain granulation tissues or inflammatory cells [3].

Currently, whether ^18^F-FDG PET or PET/CT imaging can reflect the existence of tumor cell proliferation remains controversial. Some studies found that ^18^F-FDG uptake showed a clear correlation with cell proliferation and differentiation markers [4–6], but others indicated no significant correlation between them [7–9]. Given the contradictory conclusions on this issue, we retrieved clinical studies on ^18^F-FDG uptake and cell proliferation and differentiation marker Ki-67, used meta-analysis methods to analyze the pooled data, and evaluated the correlation and the degree of correlation, in order to provide evidence-based references for clinical application.

## Methods

### Document retrieval

The EMBASE, PubMed (MEDLINE included), and Cochrane Library databases were searched by 2 researchers independently to screen for research articles on the relationship between ^18^F-FDG uptake and the expression of cell proliferation marker Ki-67 published in English language between August 1, 1994-August 1, 2014. The searching terms and strategy were "positron emission tomography OR PET OR positron emission tomography/computed tomography OR PET/CT OR PET-CT OR positron emission tomography-computed tomography" AND "^18^F-FDG OR fluorodeoxyglucose OR FDG OR ^18^FDG OR FDG-F18" AND "ki67 OR ki-67 OR Ki 67 OR mitotic index OR proliferation index OR MIB1 OR MIB-1 OR mitosis index." To minimize the loss of literatures, we conducted manual searches simultaneously as well as secondary searches for the references cited in the included articles.

### Document screening

Two reviewers independently screened the retrieved articles according to the literature inclusion and exclusion criteria. The inclusion criteria were as follows: 1) studies using PET or PET/CT imaging to analyze the relationship between tumor ^18^F-FDG uptake and Ki-67 expression; 2) studies focusing on patients with malignant tumors, or although benign tumors were included, the vast majority of tumors were malignant; 3) tumors confirmed by cytopathology or histopathology; and 4) full-length articles published in peer-reviewed journals. The exclusion criteria included the following: 1) when data or part of the data published in separate articles, the one containing the latest and the most complete data was included; 2) animal experiments, reviews, case reports, abstracts, letters, reviews, commentary, cell experiments, and lectures; 3) articles on post-treatment patients only; 4) the number of patients was fewer than 10; and 5) articles did not provide a correlation coefficient or enough data to calculate the correlation coefficient. When the 2 independent researchers had disagreement over screened articles, the inclusion was decided by 3rd investigator.

### Data extraction

The data were extracted from the included literatures by 2 investigators independently, and the extracted contents included the following: 1) basic information of the study, including the author, number of patients, tumor type, year of publication; 2) Ki-67 measurement techniques, including the specimen collection method (cell biopsy or surgery), counting method (manual or automatic counting), and labeling index (LI) calculation (Ki-67_max_ or Ki-67_mean_; LI calculated in the tumor region with highest proliferation rate is considered as Ki-67_max_, otherwise as Ki-67_mean_); 3) count information of PET or PET/CT measurement of ^18^F-FDG, including imaging equipment (PET or PET/CT), ^18^F-FDG dose, imaging agent uptake time, emission scanning time, outlining method for tumor region of interest, uptake index (SUV_max_, SUV_mean_ or other); and 4) correlation coefficient between ^18^F-FDG uptake and Ki-67 expression, including the Spearman correlation coefficient, Pearson correlation coefficient, and r^2^. If the article did not directly report the value of correlation coefficient r, r value was calculated based on the raw data or scatter plot, using the free software Engauge Digitizer and the SPSS 18.0 software. This study selected Spearman correlation coefficient for analysis. Since the Spearman correlation coefficient has already been processed by logarithmic conversion, it does not need to undergo the conversion again. The Pearson correlation coefficients were converted to Spearman correlation coefficients for further analysis [10]. The sampling of Spearman correlation coefficient is not normally distributed. Because its CI depends on the value of correlation coefficient, we converted the Spearman correlation coefficient by Fisher transformation, to obtain the z value with approximately normal distribution. The z value was then converted by inverse Fisher transformation, to obtain the Spearman correlation coefficient and related CI [11]. When there were multiple correlation coefficients calculated from several SUV or Ki-67 values, the highest value was chosen. When the 2 researchers had differences in data extraction process, a third investigator joined to form a committee to vote for a decision.

### Evaluation of the literature quality

Two investigators independently assessed the quality of the articles according to the QUADAS-2 [12], the scale table of which consists of 2 parts of contents: "risk assessment" and "practical application." The former was assessed from 4 aspects as patient selection, reference standard, index test, and flow and timing, and the latter included 3 aspects as patient selection, reference standard, and index test.

To ensure that QUADAS-2 is applicable in the present study, we set the Ki-67 detection and ^18^F-FDG PET (or PET/CT) examination as "reference test" and "index test" respectively. In this study, we chose 4 weeks as the threshold interval between PET or PET/CT examination and Ki-67 detection. When there were assessing differences between the 2 researchers, they were determined by the 3rd researcher.

### Meta-analysis

The combined correlation coefficient between ^18^F-FDG SUV and Ki-67 LI was calculated according to the values of correlation coefficient r provided in each article. Correlation coefficients were converted by the Fisher's r-to-z transformation to obtain approximately normally distributed z-values to further calculate the 95% CI. This study used a random effects model to pool and analyze. The r<0.21 indicated poor correlation; 0.21≤r<0.41 suggested average correlation; 0.41≤r<0.61 was considered moderate correlation; 0.61≤r<0.81 meant significant correlation; and r≥0.81 indicated strong correlation [13]. The existence of publication bias was assessed using the Begg's funnel plot and Begg's statistics.

The heterogeneity among r values of different studies was tested using Chi-square test and inconsistency index methods at the testing level of a = 0.05. The significance of heterogeneity was presented as *P* and I^2^ values, and *P*<0.05 or I^2^>50% indicated the presence of significant heterogeneity [14]. In case of the existence of heterogeneity, the sources of heterogeneity were further explored by sensitivity analysis.

Subgroup analysis was grouped according to factors as the following: 1) tumor type, 2) Ki-67 LI measurement method (Ki-67_max_ or Ki-67_mean_), 3) pathology collection methods (surgery or biopsy), 4) application of PET or PET/CT, and 5) SUV index (SUV_max_, SUV_mean_, etc.).

Statistical analysis was performed using STATA 11 software package (Stata Corporation, College Station, TX, USA). *P*<0.05 was considered statistically significant.

## Results

### The results of literature search and screening

**Fig 1:**
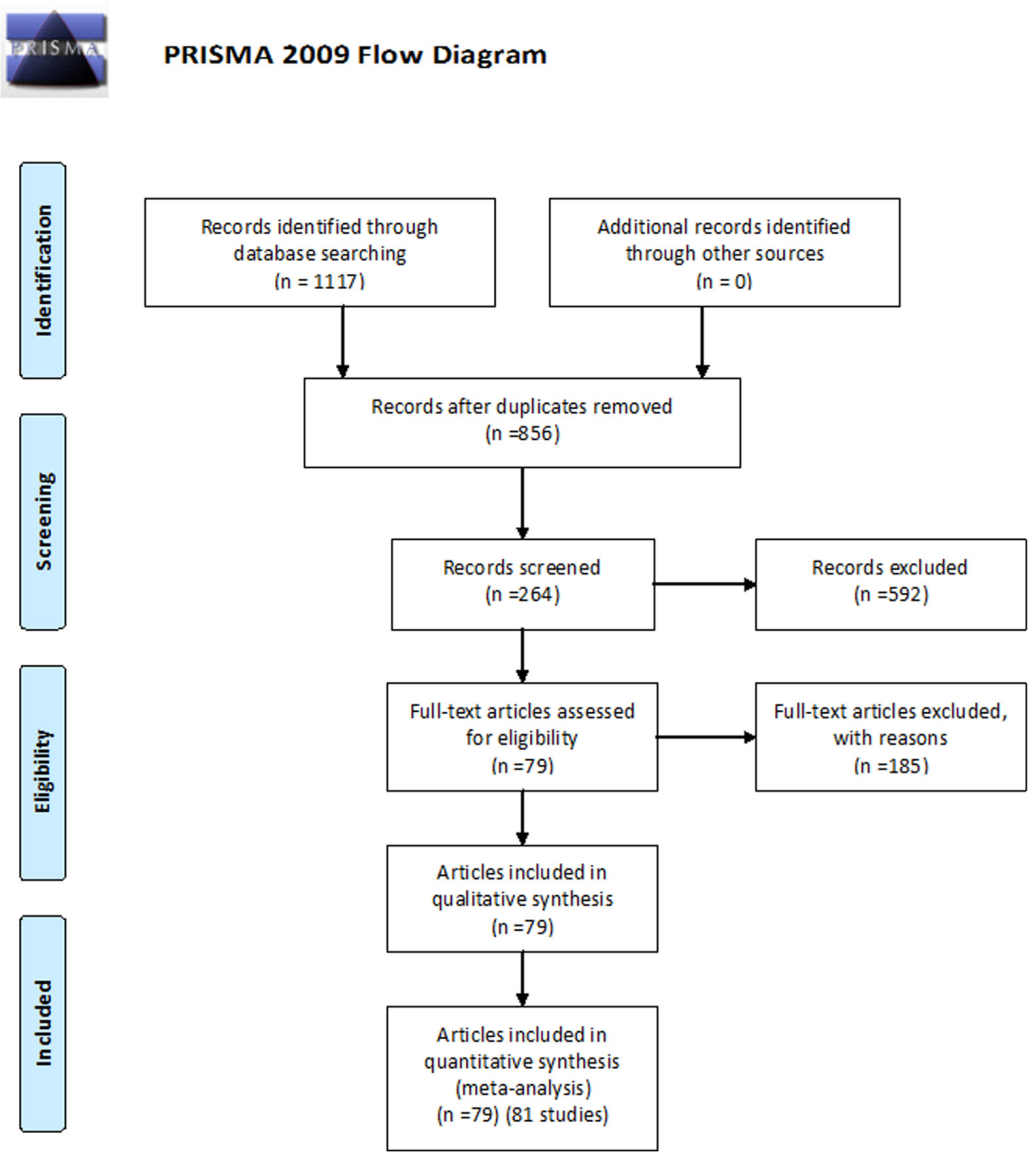
Flow diagram of study selection.

A total of 1117 related articles were retrieved from the initial search (Fig 1). After removing the repetitive ones, the 856 remaining abstracts were further screened according to the inclusion and exclusion criteria, and 264 possibly eligible articles underwent full-text screening. A total of 185 articles were eventually excluded for reasons as the following: 1) the article did not involve the evaluation of the relationship between Ki-67 expression and ^18^F-FDG uptake (n = 134); 2) the number of cases studied was fewer than 10 (n = 17); 3) the original data in the article failed to generate the correlation coefficient values (n = 27); 4) part of the data in the study appeared in other articles (n = 6); and 5) most of the cases studied were benign tumors (n = 1).

Ultimately, 79 articles were included in the present study [15–93]. One of them contained studies on 3 types of tumors and was counted as 3 studies. Therefore, the present analysis included a total of 81 studies.

### Basic information of the included studies

All included articles were published between 2001–2014, involving a total of 3242 patients, and the median cases enrolled in each individual study were 30 (range, 10–213). In 3 studies, some patients received multiple examinations. Therefore, the present analysis included a total of 3246 examinations. The tumor types contained in various studies are shown in S1 Table.

In all studies included, the mostly involved tumor type was lung cancer, analyzed in a total of 17 studies, followed by breast cancer and lymphoma, in 13 and 12 studies respectively. Brain tumors were analyzed in 5 studies. Other tumor types included GISTs, bone and soft tissue sarcoma, malignant melanoma, head and neck cancer, as well as esophageal, pancreatic, gastric, colorectal, thyroid, ovarian, oral, thoracic, thymic, uterine, and hepatocellular cancers.

For the measurement of Ki-67 expression, the majority of studies used surgically-acquired specimens (37 studies) and manual count (53 studies), and calculated the expression of Ki-67 in the regions with highest proliferation rate (Ki-67_max_, 29 studies).

For ^18^F-FDG scans, 39 studies used PET examination, while 35 used PET/CT; there were 6 studies used both PET and PET/CT, and 1 study did not report the instrument usage. While SUV_max_ was used to calculate r value in 52 studies, 12 and 17 studies used SUV_mean_ and other SUV values respectively to conduct the calculation (S2 Table).

### The results of QUADAS-2 assessing the quality of the included articles

**Fig 2:**
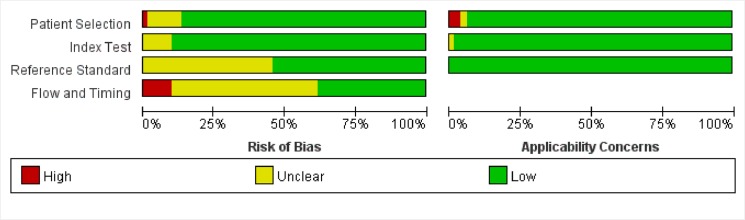
Methodological quality of all eligible studies.

As shown in Fig 2, the results of QUADAS-2 assessing the quality of the included articles indicated that the results of 11 studies showed low risk of bias in all the aspects assessed. Among all the 81 studies, 11 in the aspect of patient selection, 8 in index text, 37 in reference standard, and 50 in flow and timing displayed unknown or high risk.

Lack of an explicit description of the time interval between the reference and index tests was a problem of most studies. A total of 42 studies did not provide the time interval between ^18^F-FDG PET (or PET/CT) examination and Ki-67 measurement, and another 8 studies were assessed as high risk for having time intervals over 4 weeks.

In 44 studies, the interpretation of reference standard was clearly stated as under unknown index test, while 37 studies did not state clearly.

In addition, patients enrolled in 2 studies were with recurred tumors, while cases in another study were patients with second primary tumors. In these 2 types of patients, whether the relationship between ^18^F-FDG uptake and Ki-67 expression differs from that in patients with single primary tumor is unclear; therefore, the risk of case selection bias in the above 3 studies was considered high in the present analysis.

### The results of meta-analysis of ^18^F-FDG/Ki-67 correlation and heterogeneity test

The data provided by the finally included studies all met the standard of meta-analysis. The r value for 1 study was calculated from the provided r^2^, and the r value for another study was determined from the provided scatter plot. For another 6 studies, r values were calculated based on the provided raw data of corresponding Ki-67 and SUV.

**Fig 3:**
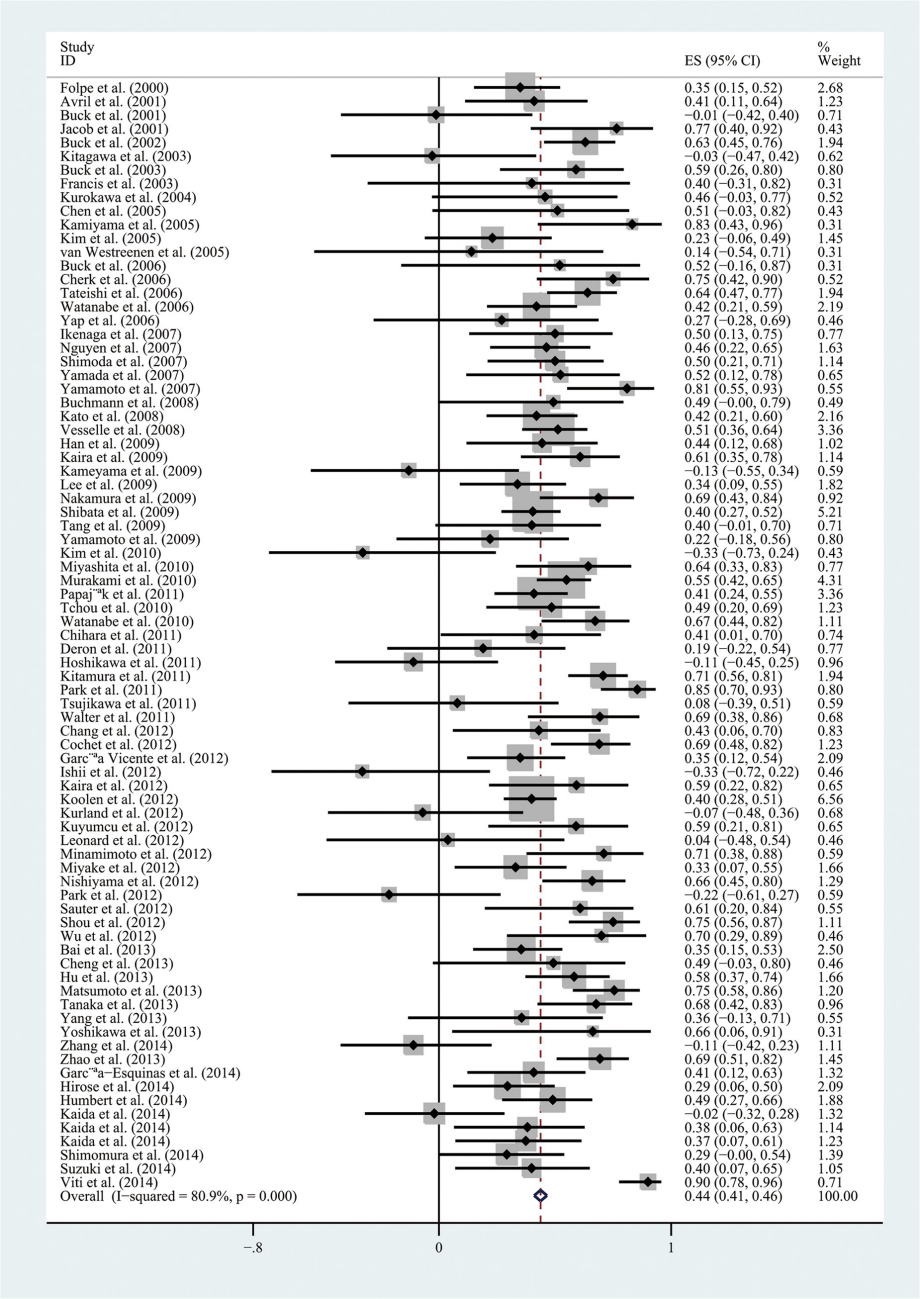
Forest plots of the summary correlation coefficient (r) with corresponding 95% CIs for the correlation between ^18^F-FDG uptake and tumor cell proliferation in all eligible studies.

**Fig 4:**
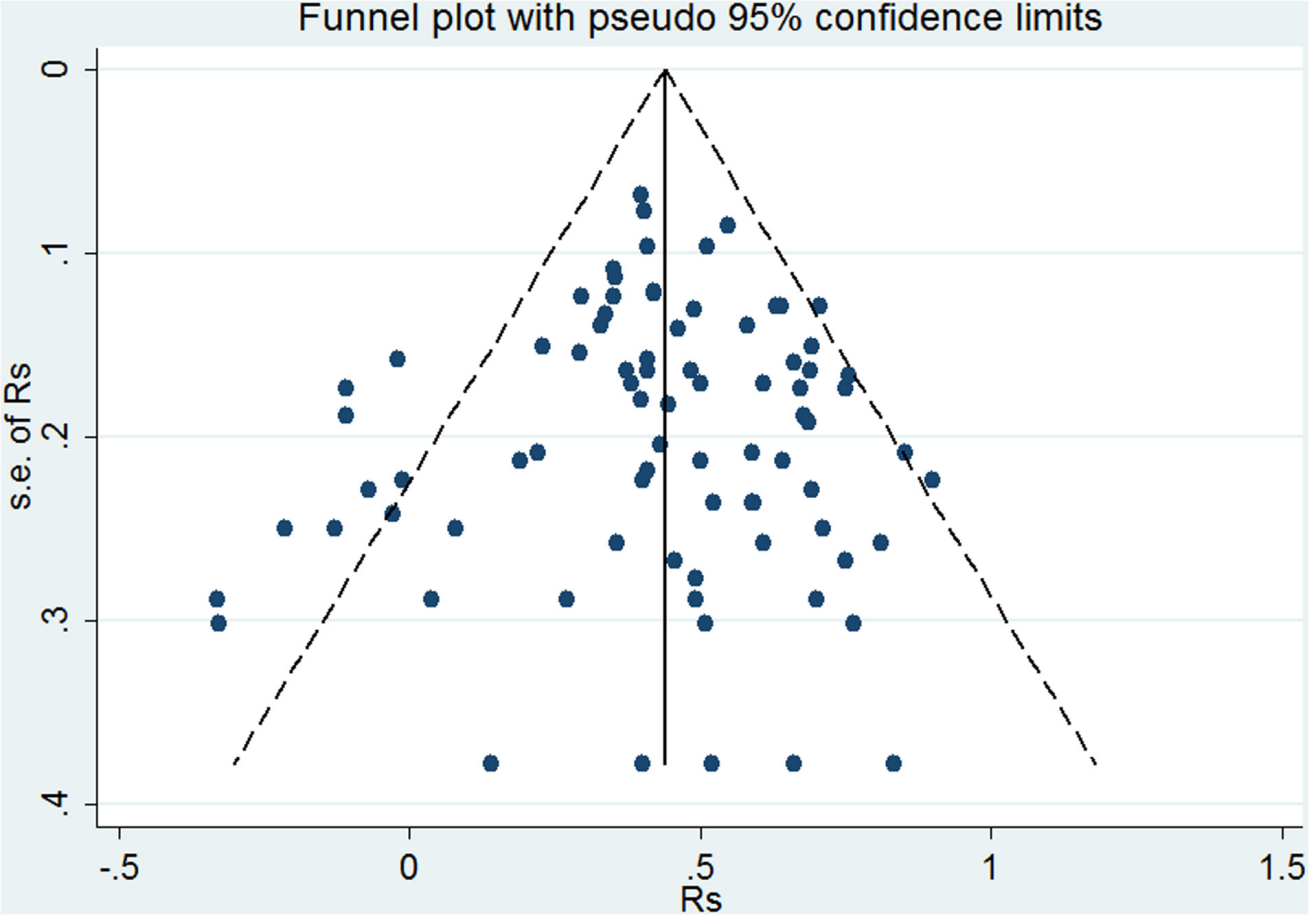
The funnel plot of the publication bias.

The combined r value finally calculated from all the included articles was 0.44 (95% CI, 0.41–0.46), but the results of heterogeneity test indicated the presence of marked heterogeneity among studies (I^2^ = 80.9%, *P*<0.01; Fig 3). We then conducted a sensitivity analysis, by excluding each article at a time to observe its effect on the final outcome, but the results showed that no individual study contributed more greatly to the total heterogeneity. The results of Begg's test indicated no significant publication deviation among the included articles (*P*>0.05; Fig 4).

**Fig 5:**
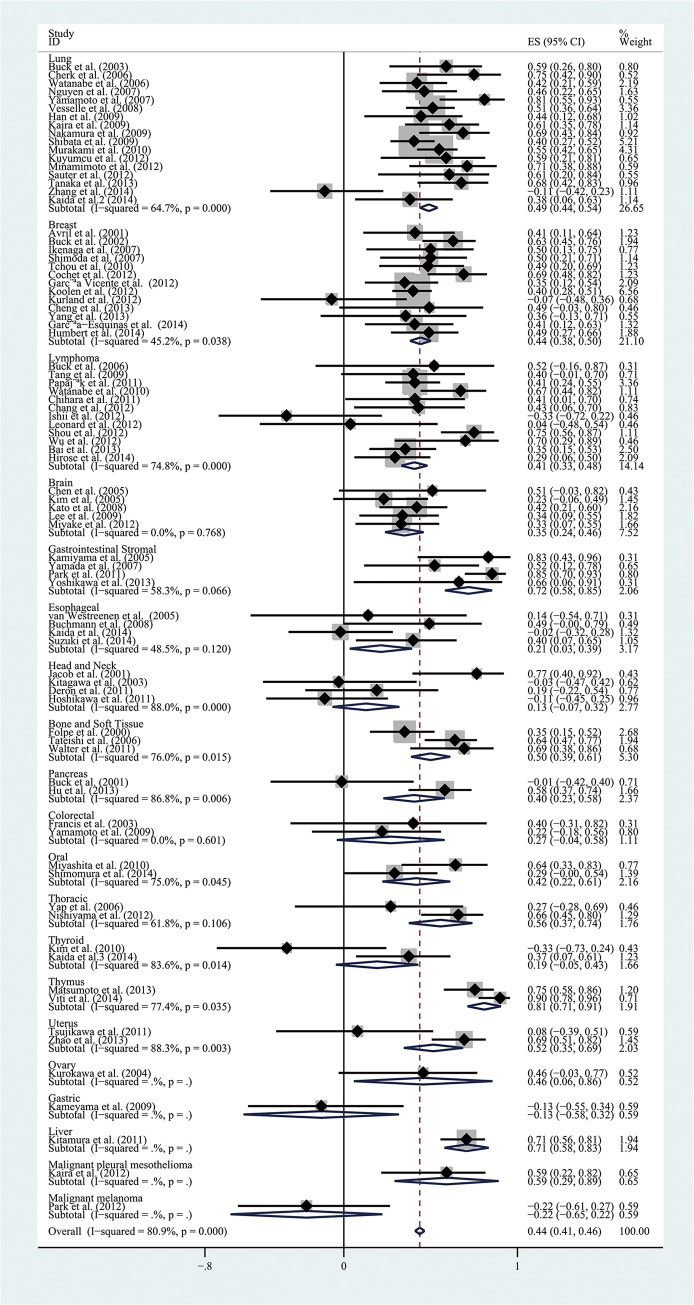
Forest plot of subgroup analysis based on cancer type.

As shown in Fig 5, subgroup analysis for tumor types showed that the combined r for the 17 studies of lung cancer was 0.49 (95% CI, 0.44–0.54; *P*<0.01), and there was significant heterogeneity among the included studies (I^2^ = 64.7%, *P*<0.01). Because the number of lung cancer is relatively larger, we further divided these studies into 4 subgroups as non-small cell lung cancer (NSCLC), adenocarcinoma, multiple tumor types, and other tumor types for analysis. The heterogeneity of NSCLC and adenocarcinoma subgroups decreased (I^2^ = 45.8%, *P*>0.05 and I^2^ = 55.6%, *P*>0.05, respectively), but the change of the combined r value showed a different trend, increased in NSCLC subgroup (r = 0.55; 95% CI; 0.48–0.62) but declined in adenocarcinoma subgroup (r = 0.48; 95% CI, 0.40–0.55). The combined r values for the 13 studies on breast cancer and the 12 studies on lymphoma were 0.44 (95% CI, 0.38–0.50; *P*<0.01; I^2^ = 45.2%, *P*<0.05) and 0.41 (95% CI, 0.33–0.48; *P*<0.01; I^2^ = 74.8%, *P*<0.01) respectively, and the latter displayed a higher heterogeneity. The combined r value for the subgroup of 5 studies on brain tumors was 0.35 (95% CI, 0.24–0.46; *P*<0.01), without significant heterogeneity (I^2^ = 0.0%, *P*>0.05). The combined r values for the 4 studies on esophageal cancer and the 4 studies on head and neck cancer were 0.21 (95% CI, 0.03–0.39; *P*<0.05; I^2^ = 48.5%, *P*>0.05) and 0.13 (95% CI, -0.07–0.32; *P*>0.05; I^2^ = 88%, *P*<0.01) respectively, and the latter exhibited a significant heterogeneity. The combined r values for the 4 studies on GISTs and the 3 studies on bone and soft tissue sarcoma were 0.72 (95% CI, 0.58–0.85; *P*<0.05; I^2^ = 58.3%, *P*>0.05) and 0.50 (95% CI, 0.39–0.61; *P*<0.01; I^2^ = 76%, *P*<0.05).

**Fig 6:**
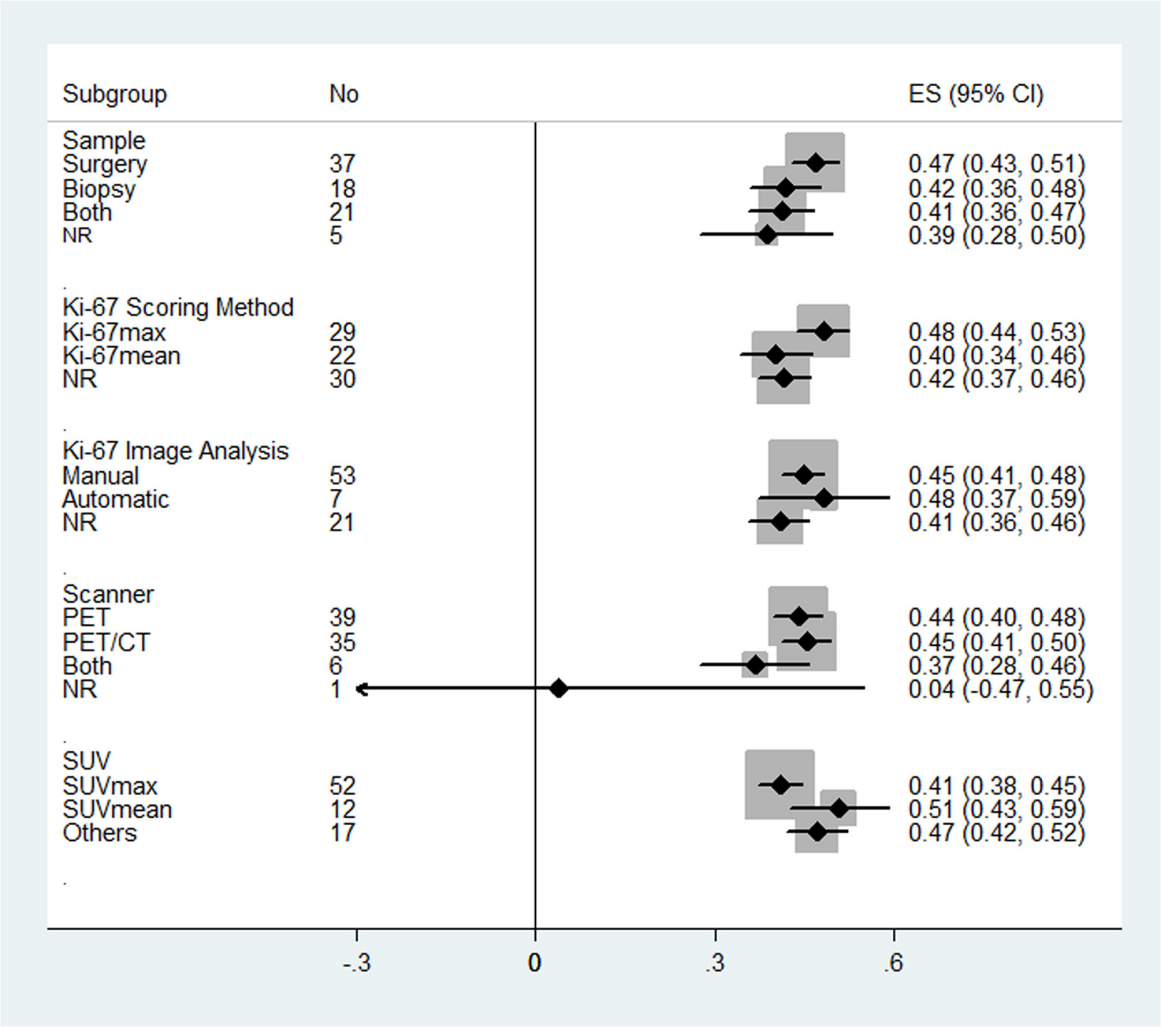
The combined correlation coefficient (r) with corresponding 95% CIs for the subgroup analysis based on sample, Ki-67 scoring method, Ki-67 imaging analysis, scanner and SUV.

In addition, the results of subgroup analysis on the different measurement methods for ^18^F-FDG SUV and Ki -67 LI are shown in Fig 6.

## Discussion

In recent years, a growing number of studies have focused on the relationship between ^18^F-FDG uptake and Ki-67 expression, which was investigated in the present study using meta-analysis methods. We analyzed the differences in their correlation among different tumor types as well as explored the differences among various methods used in ^18^F-FDG SUV and Ki-67 LI measurements. Our results showed that in cancer patients, ^18^F-FDG uptake and tumor proliferation displayed a moderate correlation. Subgroup analysis on different ^18^F-FDG SUV and Ki-67 LI measuring methods indicated that the combined r values of subgroups did not show significant changes, and neither there were significant changes in heterogeneity. However, subgroup analysis of different tumor types indicated varied degrees of correlation among different tumor types.

In this study, we used QUADAS-2 as tool to assess the quality of included studies. In most studies, the time interval between ^18^F-FDG PET (or PET/CT) imaging and the acquirement of surgical pathology specimens was not clearly stated. Among the studies included in the present analysis, most the ^18^F-FDG PET (or PET/CT) imaging was prior to the detection of Ki-67; therefore, the interpretation of ^18^F-FDG PET or PET/CT imaging results was conducted under unknown Ki-67 test results. But the vast majority of the articles did not mention whether the test results of Ki-67 were interpreted blindly. In addition, some studies did not adequately address the inclusion criteria of patients. The above problems may increase the bias of study.

In recent years, ^18^F-fluorothymidine (FLT) as a proliferation imaging agent has drawn incredible attention. ^18^F-FLT is a thymidine analogue, the uptake of which is related to the activity of thymidine kinase-1 (TK-1), the specific enzyme in the S phase of the pyrimidine salvage pathway [94]. Some studies included in the present work compared the correlations of ^18^F-FLT and ^18^F-FDG uptakes with Ki-67 expression; part of them showed that the correlation between ^18^F-FLT SUV and Ki-67 index was higher than that between ^18^F-FDG SUV and Ki-67 index [21,24], whereas others suggested that neither ^18^F-FLT SUV nor ^18^F-FDG SUV had significant correlation with Ki-67 index [27,43,48,57]. One article indicated that ^18^F-FLT uptake was not only affected by cell proliferation, but also by other mechanisms such as nucleoside transporters, etc. [43]. Another article systematically assessing the relationship between ^18^F-FLT SUV and Ki-67 expression showed a combined correlation coefficient of 0.55 [14]. Although ^18^F-FDG SUV is not an indicator directly reflecting the cell proliferation, ^18^F-FDG uptake is closely related to cell proliferation, because that tumor cell proliferation depends mainly on glycolysis for energy, and many signal transduction pathways in the process of malignant transformation of the tumor cells are regulated by glycolytic metabolism [95]. The present study showed that the combined correlation coefficient between ^18^F-FDG uptake and cell proliferation was 0.44, indicating a moderate positive correlation. This result suggested that ^18^F-FDG SUV can be used as an indicator in tumor diagnosis, to reflect the proliferation and invasiveness of tumor, and to assess the therapeutic efficacy.

In this study, we conducted subgroup analysis based on different tumor types. Previous subgroup analysis of ^18^F-FLT/Ki-67 relationship indicated that the correlation coefficient was independent of the pathological type of tumors [14]. But our findings revealed that some subgroups showed a declined heterogeneity, i.e., there were differences in the correlation between ^18^F-FDG SUV and cell proliferation among tumor types. Malignant melanoma displayed the lowest combined correlation coefficient value of -0.22 (n = 1), while the highest r (0.81, n = 2) was with the TETs. The correlation between ^18^F-FDG and Ki-67 was highly significant in TETs, significant in GISTs, moderate in lung, breast, bone and soft tissue, pancreatic, oral, thoracic, uterine, and ovary cancers, average in brain, esophageal and colorectal cancers, and poor in head and neck, thyroid, gastric and malignant melanoma tumors. One article included in the present study investigated the relationship between ^18^F-FDG uptake and various clinicopathological factors in 3 tumor types that are distinct in pathological and biological manifestations, and showed that the biological factors affecting the SUV in different pathological types of tumors varied [90]. Therefore, in different tumor types, the molecular mechanisms affecting ^18^F-FDG uptake varies, and tumor differentiation is just one of the factors, which to certain extent explains why there are differences in Ki-67/SUV relationship among different tumor types, though this issue needs to be further explored with more experiments. Currently, many clinical applications of ^18^F-FDG PET, including the localization of biopsy sites, evaluation of therapeutic efficacy, determining target region for radiotherapy, grading malignancy, etc., all are based on the assumption that ^18^F-FDG PET can accurately reflect the growth of tumor cells. If our conclusion that the correlation between Ki-67 and SUV varies in different tumor types is proven true, it can guide the correct clinical application of ^18^F-FDG PET or PET/CT in certain tumors, or the correct interpretation of the results of ^18^F-FDG examination.

This study also analyzed other possible sources of heterogeneity including the PET or PET/CT imaging technology and Ki-67 and SUV measurement methods. Our results showed that PET or PET/CT imaging technology and Ki-67 and SUV measurement methods in different institutions are different. However, results of subgroup analysis on these factors showed that no single factor was the main source leading to heterogeneity, and that the combined r values of different subgroups did not show a significant difference.

The present study has some potential limitations. First, although the numbers of patients and articles included in this study were large, they were relatively limited to a certain type of tumors, resulting in possible limitations in our inference based on the results of subgroup analysis on different tumor types. Further, the articles included in this study all directly provided correlation coefficient r values or the raw data that can be used to calculate r values. Moreover, articles reported positive or negative results without providing specific data were excluded from the present study, and this study was limited to literatures in English language, which may cause publication bias. However, the results of Begg's test showed no significant publication bias; moreover, we used a random effects model to reduce heterogeneity. Therefore, the results of the present study are reliable.

In short, although there are certain limitations in this study, our analysis indicated that in cancer patients, ^18^F-FDG uptake has a moderate positive correlation with tumor cell proliferation, and that the results of ^18^F-FDG may be used to assess tumor cell proliferation. The correlations between ^18^F-FDG and Ki-67 varied among different types of tumor, i.e., TETs and GISTs showed the more significant correlation, whereas head and neck, thyroid, gastric and malignant melanoma tumors exhibited a poor correlation; the degrees of correlation in rest the tumor types were moderate or average. Nonetheless, the results of the present work need further confirmation with large-sample, prospective studies.

## Supporting Information

S1 PRISMA Checklist PRISMA 2009 Checklist. (DOC)

S1 Table ^18^F-FDG PET scan characteristics. (DOCX)

S2 Table Ki-67 immunohistochemistry characteristics, cancer types, and r values. (DOCX)
