# Supplementary material for: Correlation between the Uptake of 18F-Fluorodeoxyglucose (18F-FDG) and the Expression of Proliferation-Associated Antigen Ki-67 in Cancer Patients: A Meta-Analysis
Source: PLoS One. 2015 Jun 3;10(6):e0129028. doi: 10.1371/journal.pone.0129028 (PMC4454667; doi:10.1371/journal.pone.0129028)
Supplement: S1 Table — (DOCX) [file pone.0129028.s002.docx]

**S1 Table.** ^18^F-FDG PET scan characteristics.

| Author | Year | Scanner | Dose (MBq) | Uptake period (min) | Emission time (min) | SUV_max_ | SUV_mean_ | Other uptake values | Delineation |
| --- | --- | --- | --- | --- | --- | --- | --- | --- | --- |
| Folpe et al. [15] | 2000 | GE Advance PET | 259-370 | 45 | NR | Yes | No | No | Manual |
| Avril et al. [16] | 2001 | Siemens ECAT 951R/31 or ECAT EXACT PET | 240-400 | 40-60 | NR | No | No | Corrected SUV_max_ | Manual |
| Buck et al. [17] | 2001 | Siemens ECAT 931 08/12 PET | 250-300 | 45 | 10 | No | Yes | No | Fixed ROI |
| Jacob et al. [18] | 2001 | Uppsala GEMS 4096 PET | 350-450 | 30 | 30 | Yes | No | No | 70% threshold |
| Buck et al. [19] | 2002 | Siemens ECAT EXACT HR+ PET | 273-481 | 45-60 | NR | No | No | T/N_max_ and T/N_mean_ | Fixed ROI |
| Buck et al. [20] | 2003 | Siemens ECAT EXACT or ECAT HR+ PET | 345-550 | 45 | 10 | Yes | Yes | No | Fixed ROI |
| Francis et al. [21] | 2003 | GE Discovery LS PET/CT | 371±24 | 47-67 | NR | No | Yes | No | Fixed ROI |
| Kitagawa et al. [22] | 2003 | GE Advance PET | 244-488 | 40 | 20 | No | Yes | No | Fixed ROI |
| Kurokawa et al. [23] | 2004 | GE Advance PET | 185 | 40 | 2 | No | Yes | No | Fixed ROI |
| Chen et al. [24] | 2005 | Siemens ECAT EXACT or ECAT HR+ PET | 148-248 | 60 | 5 | Yes | Yes | T/N | 80% threshold |
| Kamiyama et al. [25] | 2005 | Shimadzu SET 2400W PET | 275-370 | 40 | 8 | No | Yes | No | Fixed ROI |
| Kim et al. [26] | 2005 | Siemens ECAT EXACT PET | 370-555 | 40 | 20 | Yes | No | T/N | Manual |
| van Westreenen et al. [27] | 2005 | Siemens ECAT EXACT HR+ PET | 250-750 | 90 | 5 | Yes | Yes | No | 70% threshold |
| Buck et al. [28] | 2006 | Siemens ECAT EXACT or ECAT HR+ PET | 265-370 | 60 | 10 | Yes | Yes | No | Fixed ROI |
| Cherk et al. [29] | 2006 | CTI ECAT 951/31R PET or Philips Allegro PET or Philips Gemini PET | NR | NR | NR | Yes | No | No | Manual |
| Tateishi et al. [30] | 2006 | Siemens ECAT ACCEL PET | 300-370 | 60-80 | NR | Yes | Yes | No | 90% threshold |
| Watanabe et al. [31] | 2006 | Positron POSICAM.HZL m-POWER PET | 4.6/kg or 5.6/kg | 60 | 3 | No | No | (T-N)/(T+N) | Fixed ROI |
| Yap et al. [32] | 2006 | Siemens ECAT EXACT PET | 7.77/kg | 60 | 4 | Yes | No | No | Manual |
| Ikenaga et al. [33] | 2007 | GE Discovery LS PET/CT | 3.7/kg | 55-65 | NR | Yes | No | No | Manual |
| Nguyen et al. [34] | 2007 | Philips Allegro PET | 5.18/kg | 50 | NR | Yes | No | No | Fixed ROI |
| Shimoda et al. [35] | 2007 | Siemens Biograph Sensation 16 PET/CT | 4.5/kg | 60 | 3 | Yes | No | SUV_delayed_ | NR |
| Yamada et al. [36] | 2007 | GE Advance PET | 200 | 50 | NR | Yes | No | No | Manual |
| Yamamoto et al. [37] | 2007 | Siemens ECAT EXACT HR+ PET | 135±12 | 60 | 3 | Yes | No | No | Manual |
| Buchmann et al. [38] | 2008 | Siemens ECAT EXACT PET | 334±39 | 60 | 7 | Yes | No | No | Manual |
| Kato et al. [39] | 2008 | GE Advance NXi PET | 5/kg | 35 | 7 | Yes | No | T/N | Fixed ROI |
| Vesselle et al. [40] | 2008 | GE Advance PET | 259-407 | 45 | 15 | Yes | No | Corrected SUV_max_ | Manual |
| Han et al. [41] | 2009 | GE Discovery ST PET/CT | 300-400 | 40-60 | 2.5 | Yes | No | No | 90% threshold |
| Kaira et al. [42] | 2009 | Shimadzu SET 2400W PET | 5-6/kg | 50 | 8 | Yes | No | No | Manual |
| Kameyama et al. [43] | 2009 | Siemens ECAT EXACT HR+ PET | 3.5/kg | 60 | 3 | Yes | No | No | Manual |
| Lee et al. [44] | 2009 | Siemens ECAT EXACT PET or Philips Gemini PET/CT | 5.18/kg | 60 | 10 or 20 | Yes | No | T/N | Fixed ROI |
| Nakamura et al. [45] | 2009 | GE Discovery ST PET/CT | 250-300 | 60 | 3.5 | Yes | No | No | Automatical |
| Shibata et al. [46] | 2009 | Siemens ECAT EXACT Accel PET or GE Discovery ST PET/CT | 4.6/kg | 60 | 2 or 3 | Yes | No | T/N | Manual |
| Tang et al. [47] | 2009 | GE Discovery DST PET/CT | 555 | 90 | 3 | Yes | No | Corrected SUV_max_ | NR |
| Yamamoto et al. [48] | 2009 | Siemens ECAT EXACT HR+ PET | 3.5/kg | 60 | 3 | Yes | No | No | Fixed ROI |
| Kim et al. [49] | 2010 | Philips Gemini PET/CT | NR | 60 | 3 | Yes | No | No | Manual |
| Miyashita et al. [50] | 2010 | GE Discovery STE PET/CT | 5/kg | 60 | 3 | Yes | No | No | Fixed ROI |
| Murakami et al. [51] | 2010 | Siemens Biograph 16 HI-REZ PET/CT | 130-371 | 60 | 3 | Yes | No | No | Manual |
| Tchou et al. [52] | 2010 | Allegro Philips PET | NR | 63 | NR | Yes | No | No | Manual |
| Watanabe et al. [53] | 2010 | Shimadzu SET 2400 PET or Toshiba Aquiduo PET/CT | 296-414 | 60 | 2 | Yes | No | No | Manual |
| Chihara et al. [54] | 2011 | GE Discovery LS PET/CT | 4/kg | 60 | 3 | Yes | No | No | NR |
| Deron et al. [55] | 2011 | Philips Gemini PET/CT | 3.7/kg | 60 | NR | Yes | Yes | Corrected SUV_max_ and SUV_mean_ | Manual |
| Hoshikawa et al. [56] | 2011 | Siemens ECAT EXACT HR+ PET | 3.5/kg | 60 | 3 | Yes | No | No | Fixed ROI |
| Kitamura et al. [57] | 2011 | GE Advance PET | 222-370 | 50 | 3 | Yes | No | T/N | Manual |
| Papajík et al. [58] | 2011 | Siemens Biograph 16 HI-REZ PET/CT | 5.71/kg | 60 | NR | Yes | No | No | Fixed ROI |
| Park et al. [59] | 2011 | GE Advance PET | 200 | 50 | NR | Yes | No | No | NR |
| Tsujikawa et al. [60] | 2011 | GE Advance PET | 185 | 50 | 2-3 | No | Yes | No | Fixed ROI |
| Walter et al. [61] | 2011 | Siemens ECAT EXACT HR+ PET or Siemens Biograph Duo or Siemens Biograph 64 or Siemens Biograph PET | 5.55/kg | 58-114 | 4 | Yes | No | T/N | Manual |
| Chang et al. [62] | 2012 | GE Discovery ST 16 PET/CT | 370 | 50 | 4 | Yes | No | ΔSUV | Fixed ROI |
| Cochet et al. [63] | 2012 | Philips Gemini GXL PET/CT | 5/kg | 60 | 2 | Yes | No | Corrected SUV_max_ | Manual |
| García Vicente et al. [64] | 2012 | GE Discovery DSTE 16 PET/CT | 370 | 60 | 3 | Yes | No | SUV_delay_ and RI | Fixed ROI |
| Ishii et al. [65] | 2012 | Shimadzu SET 2400 PET or Toshiba Aquiduo PET/CT or Siemens Biograph 16 PET/CT | NR | NR | NR | Yes | No | No | NR |
| Kaira et al. [66] | 2012 | GE Advance NXi PET or GE Discovery PET/CT | 200-250 | 60 | NR | Yes | No | T/N | Fixed ROI |
| Koolen et al. [67] | 2012 | Gemini TF Philips PET/CT | 180-240 | 50-70 | 3 | Yes | No | No | Manual or Automatical |
| Kurland et al. [68] | 2012 | GE Advance PET or Discovery STE PET/CT | 259-370 | 60 | 7 | Yes | No | No | NR |
| Kuyumcu et al. [69] | 2012 | Siemens Biograph TruePoint PET/CT | 370 | 60 | 3 | Yes | No | No | NR |
| Leonard et al. [70] | 2012 | NR | NR | NR | NR | Yes | No | No | NR |
| Minamimoto et al. [71] | 2012 | Siemens Biograph 16 PET/CT | 370 | 60 | 2 | Yes | Yes | No | Fixed ROI |
| Miyake et al. [72] | 2012 | Siemens ECAT EXACT HR+ PET or Siemens Biograph mCT 64 PET | 147-295 | 45 | 5 | Yes | No | T/N | Manual |
| Nishiyama et al. [73] | 2012 | Toshiba Aquiduo PCA-7000B PET/CT | 370 | 55-60 | 3 | Yes | No | No | Manual |
| Park et al. [74] | 2012 | Philips Gemini GXL 6 PET/CT | 5/kg | 60 | 1.5 | Yes | No | No | semi-automatical |
| Sauter et al. [75] | 2012 | Siemens Hi-Rez Biograph 16 PET/CT | 339.6±23.2 | 58.0±3.4 | 3 | Yes | Yes | No | 50% threshold or Manual |
| Shou et al. [76] | 2012 | GE Discovery LS PET/CT | 5.55/kg | 45-60 | 15 | No | Yes | No | Manual |
| Wu et al. [77] | 2012 | GE Discovery STE 16 PET/CT | 370 | 60 | 3 | Yes | No | No | NR |
| Bai et al. [78] | 2013 | GE Discovery ST PET/CT | 4.4/kg | 60 | 3 | Yes | Yes | No | Manual |
| Cheng et al. [79] | 2013 | Siemens Biograph 16HR PET/CT | 7.4/kg | 60 | 2-3 | Yes | No | T/N | Manual |
| Hu et al. [80] | 2013 | Siemens Biograph 16HR PET/CT | 7.4/kg | 60 | 2 | Yes | No | No | Fixed ROI |
| Matsumoto et al. [81] | 2013 | Siemens ECAT ACCEL PET or GE 4096 Plus PET | 4/kg | 60 | NR | Yes | No | No | Manual |
| Tanaka et al. [82] | 2013 | Siemens Biograph 16 PET/CT | 185-240 | 60 | 3.5 | Yes | No | No | Manual |
| Yang et al. [83] | 2013 | Siemens Biograph 16HR PET/CT | 7.4/kg | 60 | 2-3 | Yes | No | No | NR |
| Yoshikawa et al. [84] | 2013 | Toshiba Aquiduo PET/CT | 3.7/kg | 60 | NR | Yes | No | No | NR |
| Zhao et al. [85] | 2013 | GE Advance PET | 185 | 50 | 2-3 | No | Yes | No | Fixed ROI |
| García-Esquinas et al. [86] | 2014 | Siemens Biograph PET/CT | 5/kg | NR | 3 | Yes | No | No | Manual |
| Hirose et al. [87] | 2014 | Philips Allegro PET | 4.44/kg | 60 | 2.5 | Yes | No | No | Manual |
| Humbert et al. [88] | 2014 | Philips CPET Plus or Philips Gemini GXL PET/CT | 2/kg or 5/kg | 60 | NR | Yes | No | Corrected SUV_max_ | Manual |
| Kaida et al. [89] | 2014 | Philips Allegro PET | 4.44/kg | 60 | 2.5 | Yes | No | No | Manual |
| Shimomura et al. [90] | 2014 | GE Discovery LS PET/CT | 3/kg | 60 | 2 | Yes | No | No | Fixed ROI |
| Suzuki et al. [91] | 2014 | GE Discovery STE PET/CT | 5/kg | 50 | 3 | Yes | No | No | Manual |
| Viti et al. [92] | 2014 | GE Discovery LS PET/CT | 330-400 | 55-70 | 4 | Yes | No | T/N | NR |
| Zhang et al. [93] | 2014 | Siemens Biograph 40 PET/CT | 5.55/kg | 60 | NR | Yes | No | No | Manual |

NR, not reported.
