# Supplementary material for: Correlation between the Uptake of 18F-Fluorodeoxyglucose (18F-FDG) and the Expression of Proliferation-Associated Antigen Ki-67 in Cancer Patients: A Meta-Analysis
Source: PLoS One. 2015 Jun 3;10(6):e0129028. doi: 10.1371/journal.pone.0129028 (PMC4454667; doi:10.1371/journal.pone.0129028)
Supplement: S2 Table — (DOCX) [file pone.0129028.s003.docx]

**S2 Table.** Ki-67 immunohistochemistry characteristics, cancer types, and r values.

| Author | Year | Cancer | No | Sample | Method | Image analysis | r_s_ |
| --- | --- | --- | --- | --- | --- | --- | --- |
| Folpe et al. [15] | 2000 | Bone and Soft Tissue (Sarcoma) | 87 | Both | Ki-67_mean_ | Manual | 0.35 |
| Avril et al. [16] | 2001 | Breast (Various) | 40 | Surgery | Ki-67_mean_ | Manual | 0.41 |
| Buck et al. [17] | 2001 | Pancreas (Various) | 23 | Surgery | Ki-67_max_ | Manual | -0.01 |
| Jacob et al. [18] | 2001 | Head and Neck (SCC) | 14 | Biopsy | Ki-67_mean_ | Manual | 0.77 |
| Buck et al. [19] | 2002 | Breast (Various) | 63 | Surgery | Ki-67_max_ | Automatic | 0.63 |
| Buck et al. [20] | 2003 | Lung (Various) | 26 | Both | Ki-67_max_ | Automatic | 0.59 |
| Francis et al. [21] | 2003 | Colorectal (Various) | 10 | Surgery | Ki-67_mean_ | Manual | 0.40 |
| Kitagawa et al. [22] | 2003 | Head and Neck (SCC) | 20 | Biopsy | Ki-67_mean_ | Manual | -0.03 |
| Kurokawa et al. [23] | 2004 | Ovary (Various) | 17 | Surgery | Ki-67_max_ | Manual | 0.46 |
| Chen et al. [24] | 2005 | Brain (Glioma) | 14 | Surgery | Ki-67_max_ | Manual | 0.51 |
| Kamiyama et al. [25] | 2005 | Gastrointestinal Stromal | 10 | Surgery | NR | Manual | 0.83 |
| Kim et al. [26] | 2005 | Brain (Glioma) | 47 | Surgery | Ki-67_mean_ | Manual | 0.23 |
| van Westreenen et al. [27] | 2005 | Esophageal (Various) | 10 | Biopsy | Ki-67_max_ | Manual | 0.14 |
| Buck et al. [28] | 2006 | Lymphoma (Various) | 10 | Biopsy | Ki-67_max_ | Manual | 0.52 |
| Cherk et al. [29] | 2006 | Lung (NSCLC) | 17 | Both | NR | Manual | 0.75 |
| Tateishi et al. [30] | 2006 | Bone and Soft Tissue (Sarcoma) | 63 | Both | Ki-67_max_ | Manual | 0.64 |
| Watanabe et al. [31] | 2006 | Lung (Stage IA adenocarcinoma) | 71 | NR | NR | NR | 0.42 |
| Yap et al. [32] | 2006 | Thoracic (Various) | 15 | Both | NR | Automatic | 0.27 |
| Ikenaga et al. [33] | 2007 | Breast (Various) | 25 | Biopsy | NR | Automatic | 0.50 |
| Nguyen et al. [34] | 2007 | Lung (NSCLC) | 53 | Surgery | NR | NR | 0.46 |
| Shimoda et al. [35] | 2007 | Breast (Various) | 37 | Surgery | Ki-67_mean_ | Manual | 0.50 |
| Yamada et al. [36] | 2007 | Gastrointestinal Stromal | 21 | Biopsy | Ki-67_mean_ | Manual | 0.52 |
| Yamamoto et al. [37] | 2007 | Lung (NSCLC) | 18 | Surgery | Ki-67_max_ | Manual | 0.81 |
| Buchmann et al. [38] | 2008 | Esophageal (SCC) | 16 | Surgery | Ki-67_mean_ | Manual | 0.49 |
| Kato et al. [39] | 2008 | Brain (Glioma) | 70 | Surgery | Ki-67_max_ | Manual | 0.42 |
| Vesselle et al. [40] | 2008 | Lung (NSCLC) | 109 | Both | Ki-67_max_ | Manual | 0.51 |
| Han et al. [41] | 2009 | Lung (NSCLC) | 33 | Surgery | Ki-67_max_ | Manual | 0.44 |
| Kaira et al. [42] | 2009 | Lung (NSCLC) | 37 | Surgery | Ki-67_max_ | Manual | 0.61 |
| Kameyama et al. [43] | 2009 | Gastric (Various) | 19 | Both | Ki-67_max_ | Manual | -0.13 |
| Lee et al. [44] | 2009 | Meningioma | 59 | Surgery | NR | NR | 0.34 |
| Nakamura et al. [45] | 2009 | Lung (NSCLC) | 30 | Surgery | Ki-67_mean_ | Manual | 0.69 |
| Shibata et al. [46] | 2009 | Lung(Adenocarcinoma) | 169 | Surgery | NR | Manual | 0.40 |
| Tang et al. [47] | 2009 | Lymphoma (FL) | 23 | Both | Ki-67_mean_ | Manual | 0.40 |
| Yamamoto et al. [48] | 2009 | Colorectal (Various) | 26 | Surgery | Ki-67_max_ | Manual | 0.22 |
| Kim et al. [49] | 2010 | Thyroid (PTC) | 14 | Surgery | Ki-67_mean_ | Manual | -0.33 |
| Miyashita et al. [50] | 2010 | Oral (SCC) | 25 | Surgery | Ki-67_max_ | NR | 0.64 |
| Murakami et al. [51] | 2010 | Lung(Adenocarcinoma) | 140 | Surgery | Ki-67max | Manual | 0.55 |
| Tchou et al. [52] | 2010 | Breast (Various) | 40 | Both | NR | NR | 0.49 |
| Watanabe et al. [53] | 2010 | Lymphoma (NHL) | 36 | Biopsy | Ki-67_mean_ | NR | 0.67 |
| Chihara et al. [54] | 2011 | Lymphoma (DLBCL) | 24 | Biopsy | NR | NR | 0.41 |
| Deron et al. [55] | 2011 | Head and Neck (SCC) | 25 | Both | Ki-67_mean_ | Automatic | 0.19 |
| Hoshikawa et al. [56] | 2011 | Head and Neck (SCC) | 31 | Both | Ki-67_max_ | Manual | -0.11 |
| Kitamura et al. [57] | 2011 | Liver (HCC) | 63 | Surgery | NR | Manual | 0.71 |
| Papajík et al. [58] | 2011 | Lymphoma (NHL) | 109 | Biopsy | Ki-67_max_ | Manual | 0.41 |
| Park et al. [59] | 2011 | Gastrointestinal Stromal | 26 | Surgery | NR | NR | 0.85 |
| Tsujikawa et al. [60] | 2011 | Uterus (Endometrial carcinoma) | 19 | Surgery | NR | NR | 0.08 |
| Walter et al. [61] | 2011 | Bone and Soft Tissue (Sarcoma) | 22 | NR | NR | Automatic | 0.69 |
| Chang et al. [62] | 2012 | Lymphoma (NHL) | 27 | Biopsy | NR | Manual | 0.43 |
| Cochet et al. [63] | 2012 | Breast (Various) | 40 | Biopsy | Ki-67_mean_ | Manual | 0.69 |
| García Vicente et al. [64] | 2012 | Breast (Various) | 68 | Both | NR | NR | 0.35 |
| Ishii et al. [65] | 2012 | Lymphoma (ENKL) | 15 | NR | NR | NR | -0.33 |
| Kaira et al. [66] | 2012 | Malignant pleural mesothelioma (Various) | 21 | Both | Ki-67_max_ | Manual | 0.59 |
| Koolen et al. [67] | 2012 | Breast (Various) | 213 | Biopsy | NR | NR | 0.40 |
| Kurland et al. [68] | 2012 | Breast (Various) | 22 | Biopsy | NR | Manual | -0.07 |
| Kuyumcu et al. [69] | 2012 | Lung (PC) | 21 | NR | NR | NR | 0.59 |
| Leonard et al. [70] | 2012 | Lymphoma (MCL) | 15 | Biopsy | NR | Automatic | 0.04 |
| Minamimoto et al. [71] | 2012 | Lung (NSCLC) | 19 | Surgery | Ki-67_max_ | Manual | 0.71 |
| Miyake et al. [72] | 2012 | Brain (Glioma) | 54 | Surgery | Ki-67_max_ | Manual | 0.33 |
| Nishiyama et al. [73] | 2012 | Chest wall sarcoma | 42 | Both | Ki-67_max_ | Manual | 0.66 |
| Park et al. [74] | 2012 | Malignant melanoma | 19 | Both | NR | Manual | -0.22 |
| Sauter et al. [75] | 2012 | Lung (NSCLC) | 18 | Both | Ki-67_max_ | Manual | 0.61 |
| Shou et al. [76] | 2012 | Lymphoma (Various) | 36 | Both | Ki-67_mean_ | NR | 0.75 |
| Wu et al. [77] | 2012 | Lymphoma (DLBCL) | 15 | Biopsy | Ki-67_max_ | Manual | 0.70 |
| Bai et al. [78] | 2013 | Lymphoma (ENKL) | 81 | NR | NR | NR | 0.35 |
| Cheng et al. [79] | 2013 | Breast (ER-α positive breast cancer) | 15 | Both | NR | NR | 0.49 |
| Hu et al. [80] | 2013 | Pancreas (Various) | 54 | Surgery | Ki-67_max_ | Manual | 0.58 |
| Matsumoto et al. [81] | 2013 | Thymic epithelial tumors | 39 | Both | Ki-67_max_ | Manual | 0.75 |
| Tanaka et al. [82] | 2013 | Lung (Adenocarcinoma) | 31 | Surgery | Ki-67_mean_ | Manual | 0.68 |
| Yang et al. [83] | 2013 | Breast(Invasive ductal breast carcinoma) | 18 | Surgery | NR | Manual | 0.36 |
| Yoshikawa et al. [84] | 2013 | Gastrointestinal Stromal | 10 | Surgery | NR | NR | 0.66 |
| Zhao et al. [86] | 2013 | Uterus (Mesenchymal uterine tumor) | 47 | Surgery | NR | Manual | 0.69 |
| García-Esquinas et al. [87] | 2014 | Breast (Various) | 43 | Biopsy | NR | NR | 0.41 |
| Hirose et al. [88] | 2014 | Lymphoma (DLBCL) | 68 | Both | NR | NR | 0.30 |
| Humbert et al. [89] | 2014 | Breast (Luminal HER2-negative breast cancer) | 61 | Biopsy | NR | NR | 0.49 |
| Kaida et al. [90] | 2014 | Esophageal (SCC) | 43 | Surgery | Ki-67_mean_ | Manual | -0.02 |
|  | 2014 | Lung (NSCLC) | 37 |  |  |  | 0.38 |
|  | 2014 | Thyroid (PTC) | 40 |  |  |  | 0.37 |
| Shimomura et al. [91] | 2014 | Oral (SCC) | 45 | Biopsy | Ki-67_max_ | Manual | 0.29 |
| Suzuki et al. [92] | 2014 | Oesophageal (SCC) | 34 | Surgery | Ki-67_max_ | Manual | 0.40 |
| Viti et al. [93] | 2014 | Thymic epithelial tumor | 23 | Surgery | Ki-67_mean_ | Manual | 0.90 |
| Zhang et al. [85] | 2014 | Lung (Various) | 36 | Both | Ki-67_mean_ | NR | -0.11 |

SCC, squamous cell carcinoma; NSCLC, non–small cell lung cancer; FL, follicular lymphoma; PTC, papillary thyroid cancer; NHL, non-Hodgkin’s lymphoma; DLBCL, diffuse large B cell lymphoma; HCC, hepatocellular carcinoma; ENKL, extranodal natural killer/T cell lymphoma, nasal type; PC, pulmonary carcinoid; MCL, mantle cell lymphoma; and NR, not reported.
